# Bronchoscopic lung lavage and exogenous surfactant successfully reverse respiratory failure after severe chlorine exposure: A pediatric case report

**DOI:** 10.1002/ccr3.9302

**Published:** 2025-01-14

**Authors:** Paola Papoff, Benedetto D'Agostino, Roberto Cicchetti, Antonella Bitti, Valentina Pennetta, Elena Caresta

**Affiliations:** ^1^ Pediatric Intensive Care Unit, Department of Maternal and Child Health and Urological Sciences Sapienza University of Rome Rome Italy

**Keywords:** acute respiratory distress syndrome, bronchoalveolar lavage, chlorine, pediatric, pulmonary edema, surfactant

## Abstract

**Key Clinical Message:**

Although the symptoms of accidental chlorine inhalation are typically mild, severe exposure can result in acute respiratory distress syndrome (ARDS). We present a case of pediatric ARDS due to chlorine exposure in which lung lavage and exogenous surfactant were successful in avoiding more invasive and costly treatments.

**Abstract:**

Chlorine inhalation as a result of swimming pool chlorination accidents is relatively common. Because symptoms are typically mild (cough and bronchospasm), complications such as severe acute respiratory distress syndrome (ARDS) are identified at a late stage, which places the patient's life at risk and necessitates highly invasive interventions. We present a case of severe ARDS in a 14‐year‐old boy following accidental exposure to chlorine powder. Upon arrival in the pediatric emergency department, the patient received oxygen administration, nebulized salbutamol, and intravenous steroids because of dyspnea and cyanosis. Despite this treatment, the patient's respiratory condition deteriorated. Early intubation and lung‐protective ventilation transitorily improved hypoxemia. Given the underlying pathophysiology of chlorine lung injury characterized by surfactant dysfunction, the boy was treated with repeated bronchoscopic lung lavages followed by exogenous surfactant (Curosurf 30 mg/kg total), 1 h after intubation and subsequently after 6 and 12 h, when he achieved a significant and stable reduction in ventilatory requirements. This case study illustrates the beneficial effects of target therapy with bronchoscopic lung lavage followed by exogenous surfactant in chlorine related ARDS to prevent more invasive and costly treatments.

## Graphical abstract

Extended parenchymal opacities are present in both lungs.

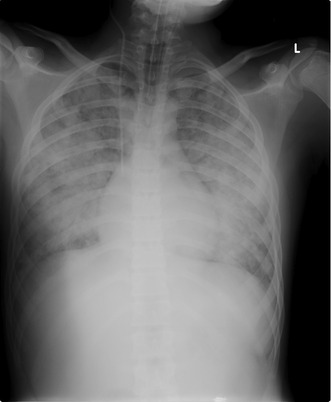

## 1 INTRODUCTION

Chlorine gas, a choking agent, was first used as a chemical weapon during World War One. In modern times, most cases of chlorine gas exposure result from swimming pool chlorination accidents, improper use of household cleaners, or, less frequently, from industrial accidents, incineration of solid chlorine products, deliberate use, as chemical warfare agents.^1^

Acute exposure to chlorine gas results in respiratory impairment, which may resolve within days or weeks, or persist as a chronic respiratory illness with asthma‐like symptoms.^2^, ^3^ The severity of lung injury from chlorine exposure depends on the duration of exposure, the gas concentration, the individual susceptibility and the water content of the exposed tissues.^4^

Low‐level acute exposure to chlorine causes irritation of the mucous membranes, which manifests as lacrimation, burning of the nose and throat, and bronchospasm. In contrast, high‐level exposure results in both airway and alveolar injury. When inhaled, chlorine dissolves in the lung epithelial lining fluid and reacts with water producing hypochlorous acid and hydrochloric acid, which have a high reactivity and oxidizing potential responsible for chlorine toxicity.^5^ As a result, injured cells release inflammatory mediators, which activate macrophages and promote vasodilation and mucus production.^6^ Vasodilation and endothelial‐epithelial barrier injury cause plasma influx into the interstitium and alveolar spaces with consequent severe pulmonary edema. Vascular leakage,^7^ inflammation, and protein oxidation contribute to dilution and inactivation of the endogenous surfactant,^8^ which ultimately leads to acute respiratory distress syndrome (ARDS).^6^ The scientific literature contains numerous reports of ARDS following chlorine inhalation, with varying severity, treatment, and outcome.^4^, ^9^, ^10^, ^11^, ^12^, ^13^, ^14^, ^15^

Treatment of chlorine exposure is mostly supportive. Removing the individual from the contaminated environment is the first step in management, with humidified oxygen provided as needed. Beta‐agonists are commonly employed to manage bronchospasm. Despite their extensive use, nebulized or intravenous corticosteroids have been the subject of considerable debate due to the lack of consistent survival benefits and concerns about superimposed infections.^1^, ^11^ Awake prone positioning combined with high‐flow nasal cannula (HFNC) oxygen therapy and non‐invasive ventilation have been successfully employed to treat hypoxemia and prevent invasive ventilation.^14^, ^16^ In the most severe cases, however, mechanical ventilation represents a life‐saving measure. Ancillary therapeutic options include intravenous or aerosolized sodium bicarbonate, intravenous ascorbic acid, free radical scavengers (e.g., N‐acetyl‐L‐cysteine, deferoxamine), sevoflurane, and others.^17^ If all these measures fail, extracorporeal membrane oxygenation (ECMO) is an effective rescue therapy.^11^

Exogenous surfactant has been used to treat ARDS of different origins^18^ due to its positive effects on surface tension, lung compliance, and lung volumes. In addition, surfactant can enhance macrophage activity, mucociliary clearance, and reduce inflammation.^19^ The use of animal models of chlorine aspiration, modeled by intratracheal instillation of hydrochloric acid, has demonstrated that the administration of poractant alfa has the potential to reduce inflammatory cytokines and oxidative stress, as well as to inhibit the formation of lung edema, with the result of an improvement in the parameters of lung function.^6^

Thus, surfactant replacement therapy has a strong pathophysiologic rationale in patients with pulmonary edema and subsequent ARDS after chlorine exposure, either to prevent the progress of lung injury or to reverse established disease. This would result in less invasive treatments and improved long‐term prognosis. However, this therapeutic approach has not yet been tested.

We report here the case of a 14‐year‐old boy who developed severe ARDS after accidentally inhaling powdered chlorine, who was successfully treated with early bronchoscopic lung lavages followed by surfactant replacement therapy. We detail the clinical manifestations of severe chlorine intoxication and the associated therapeutic interventions. The objective is to facilitate the early identification and management of patients with severe forms of this condition and to propose the use of an innovative and effective treatment strategy to prevent more invasive and costly treatments.

## 2 CASE PRESENTATION

S.M., a previously healthy 14‐year‐old boy born at 34‐week gestation, had no history of asthma, atopy, or other respiratory disorders nor did he have a history of illicit drugs, alcohol, or smoking. On a hot summer afternoon, he and his father decided to clean their swimming pool. The father was wandering off to pick up the necessary items for the cleaning, when the boy discovered the boxes of chlorine hidden in the bushes next to the swimming pool. He proceeded through the bushes and bent over to open one container of chlorine powder. Upon the cap bursting, a substantial quantity of powder was released just in front of his face, due to his position in the bush. The boy experienced a burning sensation and touched his face. Luckily, he was wearing glasses, which prevented most of the powder from reaching his eyes. His mother heard him screaming and immediately washed his face, mouth and nose, but because he began breathing heavily and appearing bluish, she called the ambulance to take him to the hospital. During transportation, he developed thoracic pain, dry cough, and bronchospasm.

On arrival at the Pediatric Emergency Department of the Umberto I Teaching Hospital in Rome, the mother reported the incident and the boy was immediately treated with oxygen, nebulized salbutamol, and steroids and intravenous methylprednisone. Peripheral oxygen saturation (SpO_2_) as determined by pulse oximetry was 80% on room air. Physical examination revealed expiratory wheezes, cyanosis, and signs of respiratory distress. The patient's vital signs were a heart rate of 122 bpm, a respiratory rate of 43 breaths per minute, a refill time of 5 s, and a Glasgow Coma Scale of 15. The face and arms were visibly erythematous. The patient's respiratory condition rapidly deteriorated; an arterial blood gas analysis revealed pH 7.22, arterial partial pressure of carbon dioxide (PaCO_2_) 53 mmHg, arterial partial pressure of oxygen (PaO_2_) 72 mmHg, HCO_3_^−^ 19.6 mmol/L, and base excess −6.5 mmol/L while receiving 100% oxygen via a non‐rebreathing mask (Table 1).

**TABLE 1:** Ventilatory settings and arterial blood gas (ABG) parameters.

Location	Pediatric emergency department		Pediatric intensive care unit								
			Surfactant doses								
			I surfactant		II surfactant			III surfactant			Extubation day 6 from admission
Timing ABG parameters	On admission	1 h after HFNC	1 h after intubation	1 h after first BAL‐surfactant	6 h after first BAL‐surfactant	12 h after second BAL‐surfactant	24 h after third BAL‐surfactant	48 h after third BAL‐surfactant	72 h after third BAL‐surfactant	96 h after third BAL‐surfactant	
pH	7.22	7.26	7.29	7.36	7.17	7.39	7.49	7.49	7.48	7.48	7.49
PaCO_2_, mmHg	53	42	37	47	55	44	42	38	48	48	39
PaO_2_, mmHg	72	89	103	88	66	95	113	88	82	103	87
HCO_3_^−^, mmol/L	19.6	18.6	18.6	25.3	17.5	25.8	31.0	29.1	28.6	29.1	29.5
P/F	–	89	103	110	63	127	280	250	251	294	174
FiO_2_	~ 1.0	1.0	1.0	0.85	1.0	0.75	0.45	0.35	0.35	0.35	0.50
Body position	Supine	Supine	Supine	Prone	Prone	Prone	Prone	Prone	Supine	Supine	Supine
Mode of ventilation	Non‐rebreather mask	HFNC 40 L/min	Pressure control	Pressure control	Pressure control	Pressure control	Pressure control	Pressure control	Pressure control	Pressure control	HFNC 20 L/min
Ventilatory parameters											
PIP, cmH_2_O	–	–	33	33	33	26	23	20	19	18	–
Pplat, cmH_2_O	–	–	26	28	28	24	–	–	–	–	–
PEEP, cmH_2_O	–	–	10	10	15	15	12	11	9	8	–
VT, mL	–	–	412	450	400	430	458	430	450	440	–

Abbreviations: BAL, bronchoalveolar lavage; FiO_2_, fractional inspired oxygen; HFNC, high flow nasal cannula; P/F, PaO2/FiO2; PEEP, positive end expiratory pressure; PIP, peak inspiratory pressure; Pplat, plateau pressure; VT, tidal volume.

## 3 TREATMENT

Given that the patient's level of consciousness was normal, the emergency pediatrician determined that HFNC oxygen therapy would be the optimal choice for respiratory failure. When the pediatric intensivist was called for difficult venous access, the patient was immediately transferred to the intensive care unit and intubated using a video laryngoscope (C‐MAC Video Laryngoscope, Karl Storz, Tuttlingen, Germany), which showed mucosal erythema and laryngeal edema. A chest x‐ray confirmed the suspicion of pulmonary edema (Figure). Although the boy was deeply sedated for mechanical ventilation (Servo‐U® mechanical ventilator, Getinge), muscle relaxants were soon required because of excessive respiratory drive, which could worsen pulmonary edema. The boy was ventilated according to the ARDS‐net protocol with a plateau airway pressure of <30 cmH_2_O and a tidal volume (VT) of 6 mL/kg (predicted body weight). He was placed in prone position to improve gas distribution. The ventilator was set in pressure‐controlled mode with 10 cmH_2_O of positive end‐expiratory pressure (PEEP), 33 cmH_2_O of peak pressure and a square pressure wave. The resulting VT was 412 mL (6.33 mL/kg). PEEP and fractional inspired oxygen were adjusted according to PaO_2_ levels. After intubation, one significant feature was the copious secretions from the endotracheal tube (nearly 1 L in the first hour), which required frequent suctioning. A flexible bronchoscopy (Pentax 4.9 mm Ø) via the endotracheal tube was performed to assess for visible airway injury. Findings were significant for areas of mucosal pallor and regions of hyperemic mucosa after foamy secretions were aspirated (Figure 2A,B). Selective washing was undertaken to remove chlorine residuals and inflammatory mediators from the lung epithelium and surfactant (poractant alfa, Curosurf®, Chiesi, Parma, Italy) was administered soon after. Prior to the procedure, the patient was given an extra dose of muscle relaxant to prevent surfactant to build‐up along the tube and obstruct it. Manual ventilation was performed during and after instillation. Because after the administration of surfactant the airway resistance often increases and the volume reduces, the peak pressure was transitorily increased, and a rapid endotracheal tube aspiration was allowed. The potential risks associated with surfactant administration, including pulmonary hemorrhage, air leaks, pneumothorax, increased airway resistance, hypoxemia, and bradycardia, were carefully considered. In order to mitigate the aforementioned complications, the following precautions were taken: manual ventilation with 100% oxygen through a ventilatory bag connected to one end of a catheter mount, with the other end connected to the endotracheal tube; the elbow port of the mount was left open to permit the passage of the bronchoscope while simultaneously providing manual ventilation. After lung toilet, surfactant (720 mg diluted in 50 mL of 0.9% saline, 14.4 mg/mL) was rapidly instilled through the bronchoscope suction channel in several aliquots in the principal bronchi using a 20 mL syringe filled with 15 mL diluted surfactant and nearly 10 mL of air to empty the dead space of the suction channel. Between the two lungs, the bronchoscope was retracted to allow for manual ventilation. Dilution of surfactant was established to administer a total dosage of 20–30 mg/kg. For the off‐label use of surfactant, written informed consent was obtained from the patient's parents and authorization was granted by the hospital's drug committee. The result of this first surfactant instillation was a slight improvement of oxygenation (Table 1) and a decrease in the frequency of tracheobronchial aspirations. A few hours after the first surfactant instillation, oxygenation began to worsen, and PEEP was increased to 15 cmH_2_O. A chest radiograph showed bilateral whiteout (Figure 3). Bronchoscopic lung toilet was repeated followed by surfactant administration using the same technique. After 12 h a chest‐x‐ray was repeated, which showed slight lung improvement but still consolidation areas (Figure 4). To decrease PEEP and avoid hyperdistension of the recruited areas, lung lavage was repeated followed by surfactant administration. After this treatment oxygenation improved markedly and PEEP was gradually decreased (Table 1). Although the parameters of respiratory mechanics are not routinely reported in the clinical chart, it is possible to assume the improvement in lung compliance after surfactant by the reduction of plateau pressure in the face of a stable VT. Conversely, the reduction in airway resistance can be approximated by the reduced difference between peak pressure and plateau pressure over time. During the initial hours of admission, the patient exhibited oliguria and high fever. Boluses of fluids were used to treat hypotension and hypovolemia and low dose noradrenaline was added to stabilize the hemodynamics. Metabolic acidosis treated with bicarbonate infusion. During the period of endotracheal intubation, a state of general inflammation was found with activation of coagulation. Admission cultures of blood, urine, and bronchoalveolar lavage fluid were all negatives. Heart contractility was normal. The boy received intravenous ceftriaxone (2 g daily) and dexamethasone (8 mg twice a day). After 4 days of mechanical ventilation bronchoalveolar lavage grew *Stenotrophomonas maltophilia* and *Candida albicans* and antibiotic treatment was modified accordingly.

**FIGURE 1:**
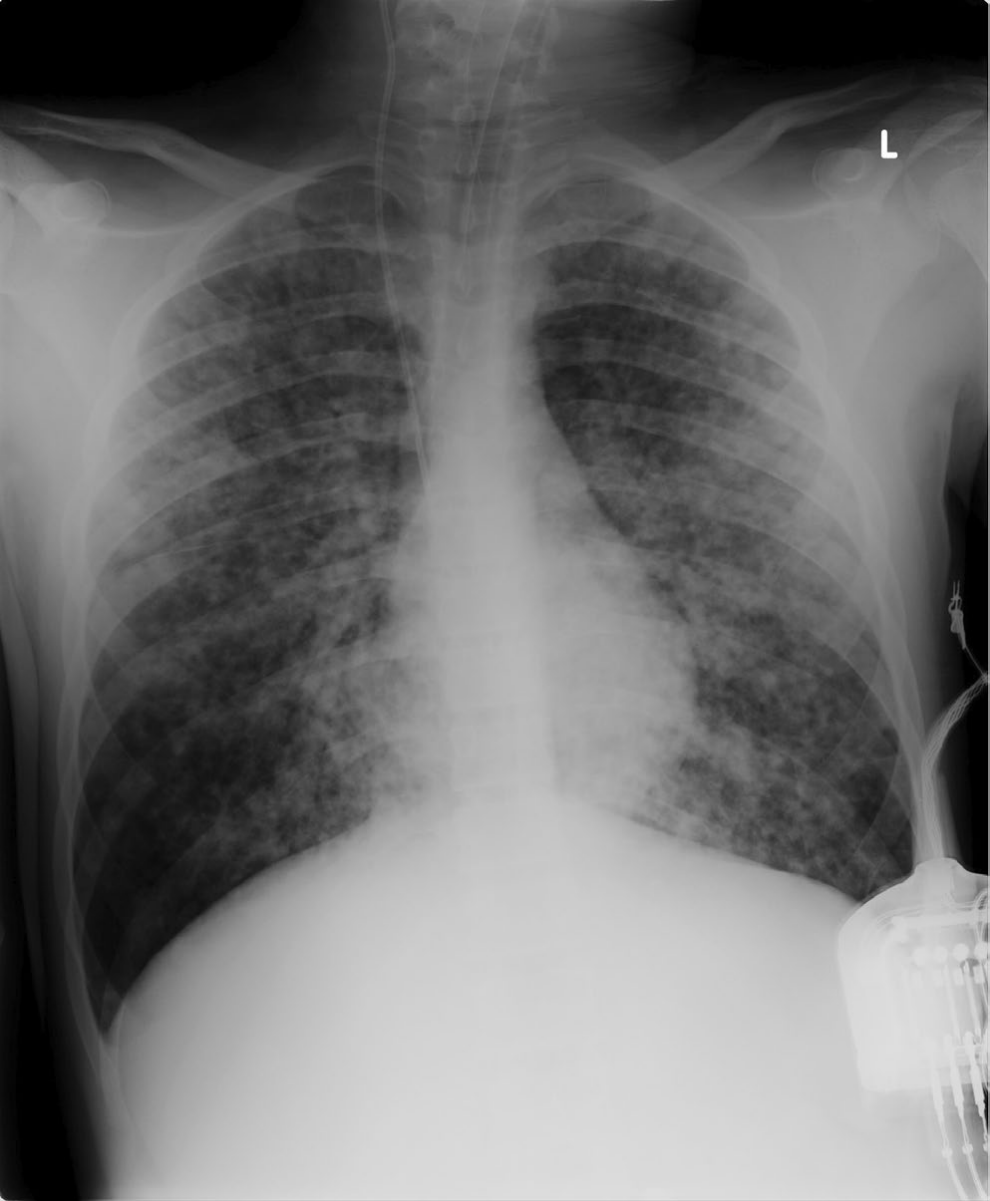
Numerous parenchymal opacities are observed in both lung areas.

**FIGURE 2:**
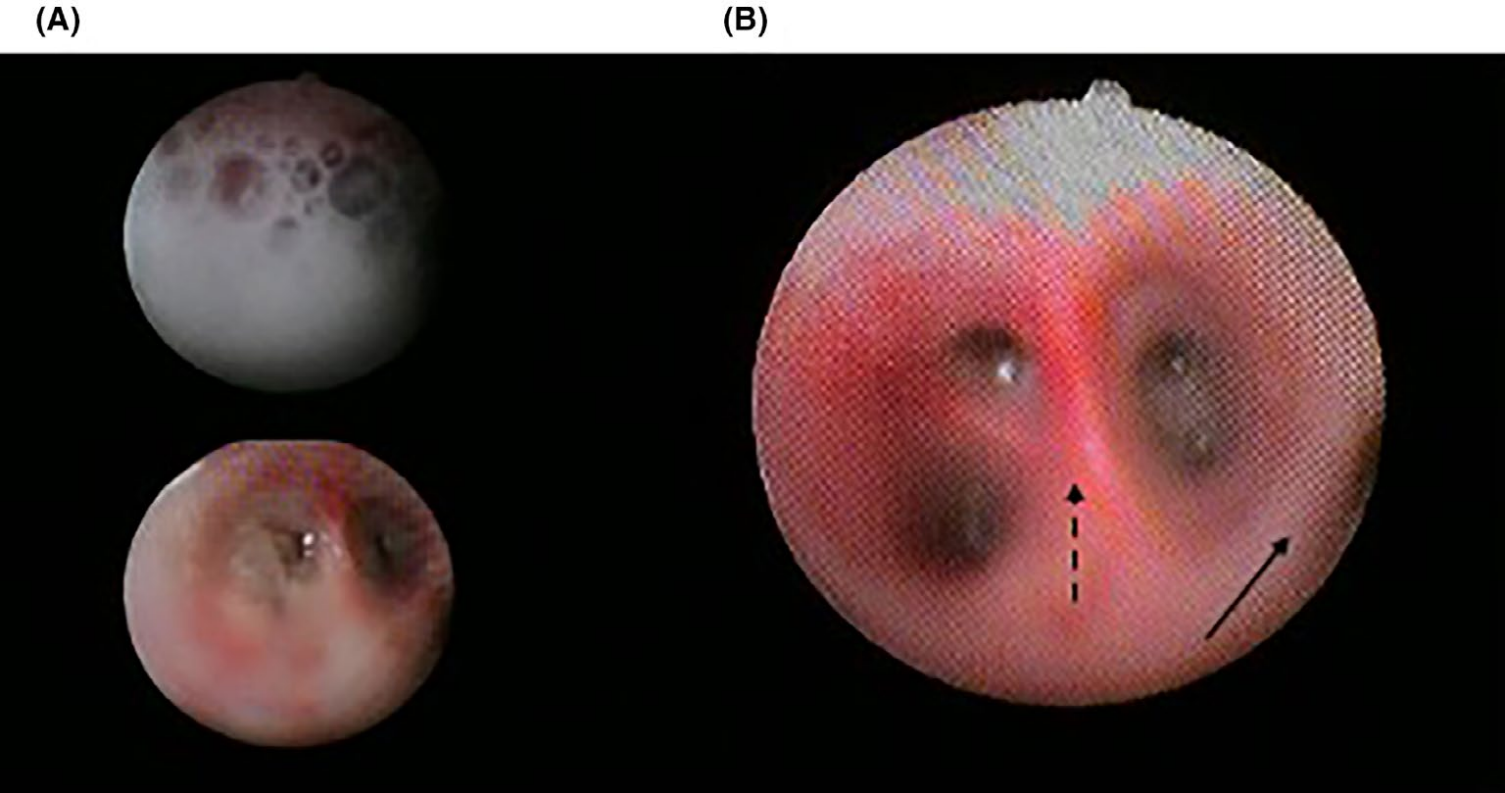
(A) Foamy secretions from bronchial branches. (B) Areas of mucosal pallor (filled arrow) and hyperemia (dashed arrow) visible after lung lavage.

**FIGURE 3:**
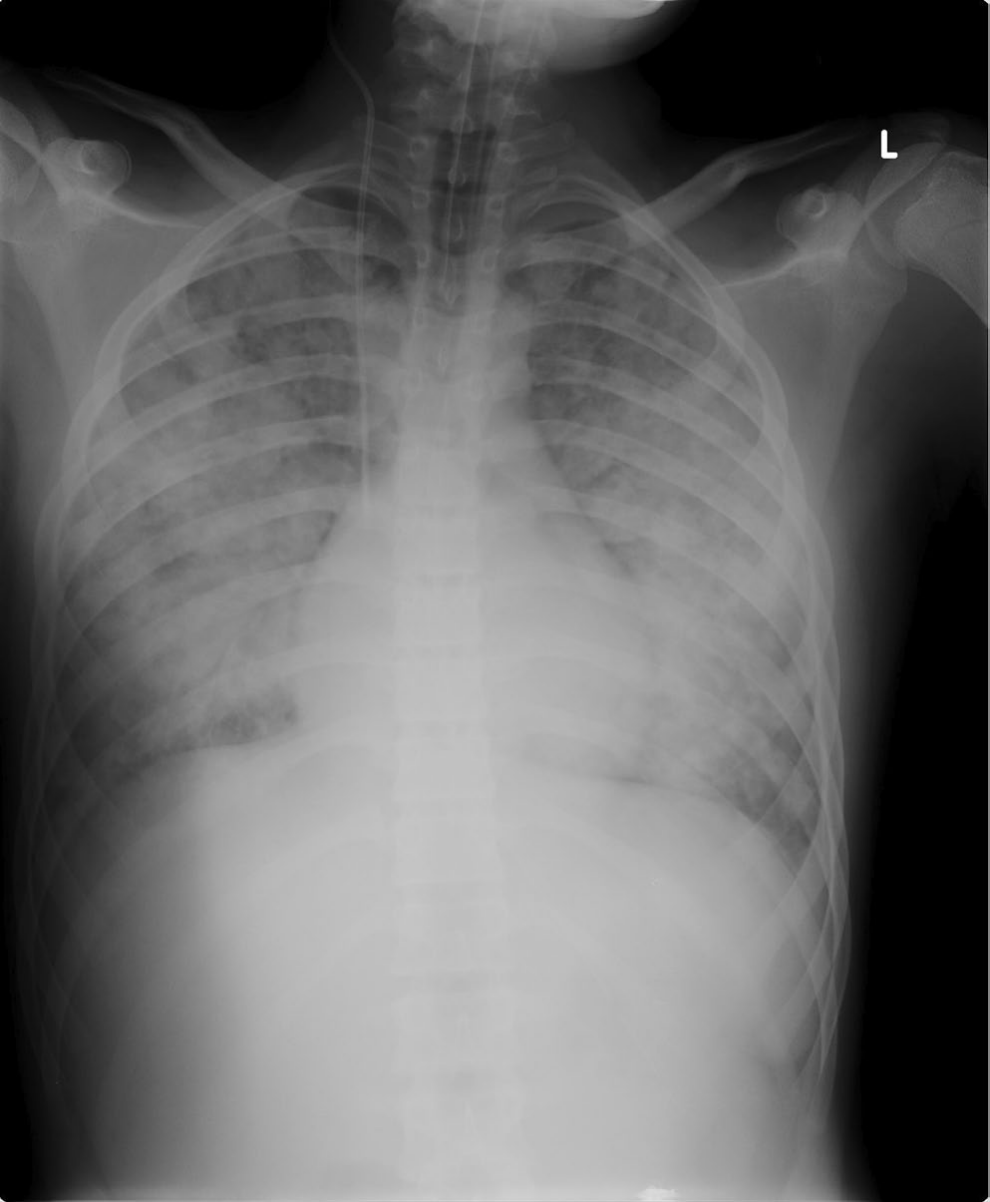
Marked increase in number and size of parenchymal opacities shown in Figure 1.

**FIGURE 4:**
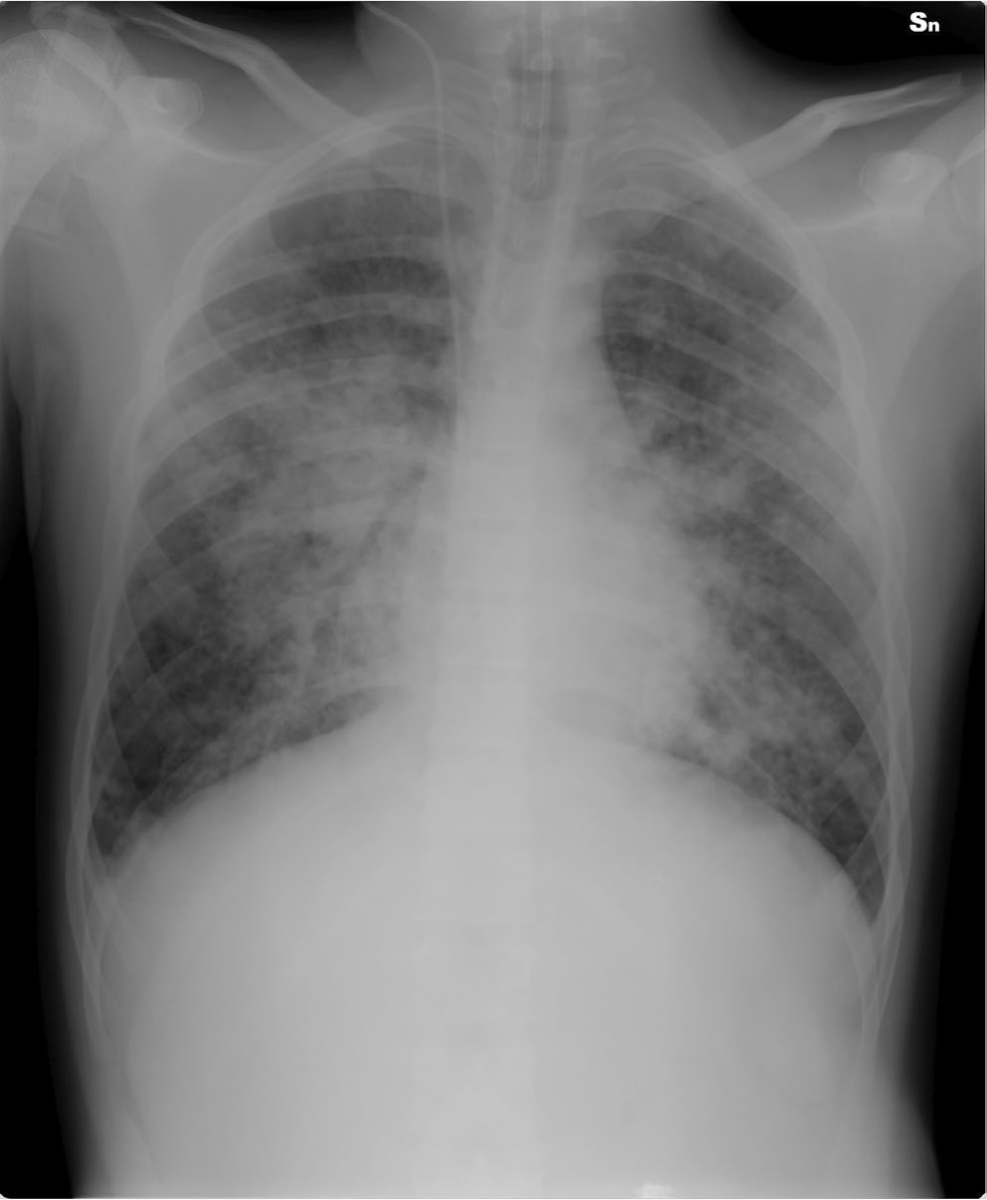
Slight reduction of the opacities shown in Figure 3. Confluent opacities with parailar distribution, bilateral, and involving the middle lobe and lingula are still present.

## 4 OUTCOME AND FOLLOW‐UP

The boy was extubated after 6 days of mechanical ventilation and discharged home after 14 days of hospital stay in good health. At follow‐up 1 year later, the patient had complete resolution of symptoms and no pulmonary complications. Spirometric studies showed normal values of forced expiratory volume in 1 s (FEV_1_) (90.6% of the predicted value and 94.1% after inhalation of 400 μg salbutamol), mid forced expiratory flow (FEF 25%–75%, 3.39 L/s and 3.55 L/s after salbutamol), and diffusing capacity divided by alveolar volume (DLCO/VA).

## 5 DISCUSSION

We describe a case of chlorine‐induced ARDS in a healthy boy, who was successfully treated with repeated bronchoscopic lung lavages followed by surfactant replacement therapy. To the best of our knowledge, only a few cases of severe ARDS due to chlorine inhalation have been reported, but none of them were treated with bronchoscopic lung lavage and exogenous surfactant to correct severe hypoxemia.

Because the most common manifestations of chlorine exposure are mucosal and airway irritation, treatment in emergency department includes nebulized salbutamol and supplemental oxygen. The approach changes in case of severe chlorine exposure, but this is rarely recognized at the beginning. Particularly in domestic accidents, it is impossible to quantify how much chlorine has been inhaled. On arrival at the emergency department, our patient presented with diffuse wheezing on auscultation, profound hypoxemia, tachycardia, severe dehydration, and vasoconstriction, as seen in pulmonary edema or ARDS at an early stage. The emergency pediatrician failed to recognize the severity of the disease due to the patient's alertness and cooperation. Consequently, standard treatment with inhaled salbutamol and steroids was initiated unsuccessfully. Targeting the pathophysiology of the resulting lung disease is essential for the management of severe chlorine inhalation. As a first step, it is crucial to acknowledge whether pulmonary edema and subsequent ARDS has developed or if airway obstruction is the cause of respiratory insufficiency. If pulmonary edema has manifested, small fluid resuscitation and diuretics are recommended. However, even in this case, a volume replacement strategy may be necessary to reverse hypotension and oliguria resulting from fluid loss into the alveolar space and high PEEP levels may help remove fluids from the alveoli. The importance of fluid resuscitation is highlighted by the report of Heidemann et al. who described of a 12‐year‐old boy who accidently inhaled chlorine.^20^ Initially, his fluid intake was restricted to prevent further deterioration of lung disease. Subsequently, hypotension and vasoconstriction necessitated the administration of additional fluid boluses. The boy was then mechanically ventilated, and a high PEEP helped to resolve pulmonary edema.

Following the onset of pulmonary edema,^21^ there is a decline in surfactant function,^22^ which results in airway collapse and subsequent respiratory dysfunction. Recognizing ARDS at an early stage and provide surfactant replacement therapy after lung toilet may have a crucial role to avoid potentially unnecessary invasive and costly therapeutical approaches.^11^ We performed a bronchoscopy soon after intubation with the objective of investigating the state of the airway. This decision proved correct, as it led to a better understanding of the severity of lung injury and the potential for improvement through lung lavages followed by surfactant replacement. We chose surfactant over other possible treatments such as sevoflurane or vitamin C, free radical scavengers (e.g., N‐acetyl‐L‐cysteine, deferoxamine), and parenteral sodium nitrate preparations, because of the restrictive nature of the respiratory insufficiency and the rapidly evolving respiratory failure.

A review of case reports using ECMO as a rescue treatment revealed the absence of targeted therapies for chlorine lung injury. It is challenging to determine whether the absence of ancillary treatments influenced the outcome. The initial case study concerns a 31‐month‐old child who sustained an accidental chemical burn from chlorine bleach and developed ARDS. He was intubated and ventilated; after a few days ARDS worsened, the patient experienced a cardiac arrest. He was placed on ECMO, but he ultimately died of neurologic complications.^15^ Another illustrative case is that of an 11‐year‐old girl who was inadvertently exposed to chlorine fumes during disinfection.^23^ The patient presented to the emergency department with severe respiratory insufficiency. After 3 days, she was intubated and ECMO was initiated due to an increase in ventilator settings. The patient ultimately recovered following the resolution of several challenging complications.^11^

A critical point determining the efficacy of surfactant therapy in ARDS is the association with strategies that can protect surfactant against inactivation, such as using direct inhibitors or reduction of inflammation and oxidation, and surfactant lung lavages.^23^ We opted for extensive lung lavage to remove toxic substances from the lung surface and then replace inactivated surfactant.

Although the administration of exogenous surfactant has a well‐founded therapeutic rationale for these patients, the existing literature is limited to a small number of experimental studies.^6^, ^24^, ^25^, ^26^ In the context of human studies on toxic inhalants, there are only cases of surfactant administration following hydrocarbon aspiration, severe burns, and near‐drowning with sand aspiration.^27^, ^28^, ^29^ Other toxic compounds that contain methyl isocyanate, sulfur mustard, or fluorescin (e.g., waterproofing spray) may also cause lung injury following inhalation.^30^, ^31^, ^32^ Although the mechanisms of cell injury produced by these compounds differ from that of chlorine, there may be some overlap in the resulting lung pathology, which could benefit from surfactant administration.

We preceded surfactant administration by selective lung lavage. Although lung lavage may appear to be a harmless procedure, several investigators have demonstrated detrimental results,^24^, ^25^ such as a loss of surface‐active material from the lung with a resultant fall in alveolar stability. Therefore, extensive lung toilet should be always followed by surfactant replacement, provided that lavage is not performed with surfactant‐diluted saline. In experimental studies on rats suffering from respiratory distress syndrome caused by chloridic acid aspiration, Eijking and colleagues, demonstrated that removal of edema fluid from the alveolar space with a diluted surfactant suspension improves the efficacy of exogenous surfactant in restoring blood gases, whereas surfactant instillation without prior lavage did not restore lung function.^26^ The best way to treat these animals appeared to be either to lavage the lungs with saline, directly followed by intratracheal instillation of a high dose of surfactant, or to lavage the lungs with a diluted surfactant suspension. In clinical practice, bronchial toilet followed by surfactant treatment has been used in children following sand aspiration and respiratory failure.^27^, ^28^ Similarly to previous experience, we have performed therapeutic bronchoscopic lung lavage with saline alone and used all the available surfactant for subsequent restoration. The dose of surfactant approved by the hospital drug committee was lower (20–30 mg/kg) than the current recommended dose for pediatric ARDS (50 mg/kg in two aliquots).^29^ It is likely that a higher dose (100 mg/kg) would have produced a more pronounced effect and that multiple doses would have been unnecessary.

## 6 CONCLUSION

In conclusion, emergency physicians should be aware of the rapid onset of pulmonary edema and subsequent ARDS resulting from severe chlorine exposure. Early airway management and optimal ventilation strategies are critical to improve outcome in these cases. If mechanical ventilation is insufficient to correct hypoxemia, bronchoscopic lung lavages followed by surfactant replacement may improve oxygenation and prevent progression into established ARDS. Nevertheless, further studies are needed before exogenous surfactant can be implemented into the treatment protocols for severe chlorine intoxication.

## AUTHOR CONTRIBUTIONS

**Paola Papoff:** Conceptualization; data curation; writing – original draft; writing – review and editing. **Benedetto D'Agostino:** Conceptualization; data curation; writing – original draft; writing – review and editing. **Roberto Cicchetti:** Data curation; investigation; writing – original draft; writing – review and editing. **Antonella Bitti:** Data curation; writing – review and editing. **Valentina Pennetta:** Data curation; writing – review and editing. **Elena Caresta:** Supervision; writing – original draft; writing – review and editing.

## FUNDING INFORMATION

This research did not receive any specific funding.

## CONFLICT OF INTEREST STATEMENT

The authors have no conflicts of interest.

## CONSENT

Written informed consent was obtained from the patient to publish this report in accordance with the journal's patient consent policy.

## Data Availability

Data and materials are available on reasonable request.
